# Practice of Drugging Children

**Published:** 1855-05

**Authors:** 


### THE PRACTICE OF DRUGGING CHILDREN.

We commend the following, found in the New Hampshire Journal of Medicine, to the attention of our readers, and would wish it could reach the fireside of every home and hamlet in the country, and there meet the eye and arrest the attention of every mother and nurse.

We are pained to acknowledge the fact, that excessive drugging is not confined to the above. Medical men, although they may not prescribe vile nostrums, are nevertheless but too prone to administer medicines, when dietetic or hygienic measures, if resorted to and relied upon solely, might subdue and control nature in some of her excesses, or bring her back from some of her absent wanderings to duty again.

But let us look into a modern nursery room, and observe what is the routine here. The little mortal being of some eighteen months scratches its little *nasal prominence* in an unlucky moment, when the mother is looking toward it, and she (and perhaps the grandmother too) witnesses the important fact. This proves a most unfortunate and untimely movement on its part, for it is condemned without judge or jury, to the devouring rapacity of *worms*, or a large share of a bottle of Fahnestock's, McClean's, or some one else's vermifuge. *Nolens volens*, the little being must swallow it, and in addition to the repulsive nature of the dose, it is horrified with the announcement that there are "*snakes in its belly.*" Although not a single parasite has been seen in its dejections, which is the *only reliable* symptom of worms, yet it is as clear as the noon-day sun, that the child *is* afflicted with worms because, simply and solely, it has *rubbed its nose.*

Another happens to cough or sneeze to the horror of its most affectionate mother, and soon a large dose of Jayne's expectorant, or some other sovereign remedy, which will almost take out the lungs, wash them, and return them again clean and well within the thoracic cavity, is forced down its throat.

Another whines with the colic, and Godfrey's cordial, Bateman's drops, paregoric, or something else of the kind in which opium is the base, is dosed down to "*cork up*" its bowels and predispose it to diseases of the brain.

What makes this practice the more revolting is, that when it is not necessary it becomes an act of foul tyranny on the part of the parent who, by brute force, compels the helpless and defenceless being to take that which should not be given under any circumstances if for no *other* reason, the very obvious one that the composition is not known. And this tyranny is perpetrated by its own mother who should not only have an affection and sympathy for her offspring, but intelligence and knowledge with it to be able to protect it from harm.

For the sake of these poor defenceless little beings, if for no other reason, these accursed nostrums should be suppressed by the potent arm of the law. If adults will guzzle down these vile cheats, to their own manifest injury, let them do it, but let the child unable, because too feeble to protect or defend itself from such outrages, be saved from them.

Let mothers learn to give their children fresh air, simple but nutricious food, encourage them to exercise, allow them to bask in the sun, for they need it as much as the plant, send them to bed early and require them to rise before the sun shall have surmounted the eastern horizon, and then to their bath, and our word for it they will have little occasion for the physician, and less occasion for patent nostrums of any kind. It is our deliberate opinion that it had been better for the health and growth of many persons if they had been raised as gypsies, and *slept in camp* all their lives, than to have been raised in close and poorly ventilated rooms where the air, by our sombre air-tight stoves, has been carbonized and rendered unfit for respiration.

Let every medical man attend to his duty, and instruct mothers and guardians on this subject, and as they become informed, these outrages will diminish.—[Eds.

Infantile Mortality.—In purusing this subject in the Boston Journal, Dr. Alcott remarks upon the pernicious influences of maternal dosing and drugging. He says: "Bad as the world is, in other departments of drugging, this is the most prolific of infantile disease, and permature death than all else, except bad cookery." He thinks that mothars are very apt to be mistaken in the constitutions and idiosyncrasies of their own children, and that they often treat scrofulous children—amounting to one-third or one-fourth of the whole—in a manner diametrically opposite to what they would have done, had they understood the nature of the case, and how the first symptoms of latent scrofula manifest themselves. The hazard of correcting them he considers in exact proportion of their ignorance, and condems in strong terms the practice of keeping a large assortment of medines at hand, as a temptation to administer much when little is needed. Physicians experience embarrasment by the irritations and inflammations of the first passages, caused by this injudicious dosing. He refers to some of the systems of quackery in vogue, but does not mention the practice of resorting to the use of secret nostrums, which is such a great and fatal evil in the South, and by which the lives of large numbers of children are sacrificed. The sale of these nostrums in Tennessee is undoubtedly the cause of greater mortality than the sale of intoxicating drinks; and yet, such is the infatuation of the people in regard to this matter, that while the advocates of temperrnce make the welkin ring with their clamor, this great evil is rarely ever mentioned in print, or spoken of in public. Mothers in this case are not only very liable to to misjudge the condition of their children, as Dr. Alcott says, but take the further risk, of administering to them drugs of which they know nothing, not even the name.

